# Three new karyotypes extend a Robertsonian fan in Ethiopian spiny mice of the genus *Acomys* I. Geoffroy, 1838 (Mammalia, Rodentia)

**DOI:** 10.3897/CompCytogen.v5i5.1252

**Published:** 2011-12-22

**Authors:** L.A. Lavrenchenko, R.S. Nadjafova, N.Sh. Bulatova

**Affiliations:** 1 A.N. Severtsov Institute of Ecology and Evolution, Russian Academy of Sciences, Moscow 119071, Russia

**Keywords:** mammalian karyotype, Robertsonian variation, *Acomys*

## Abstract

Three new karyotypes (2n=40, 44, 52) are described revealing what are probably new cryptic species of Ethiopian spiny mice. Two other diploid numbers have already been reported for the country (2n=36 and 68) and, overall, the five known karyotypic forms constitute a common lineage differentiated by a Robertsonian process. Such arrays of karyotypic forms are known as a ‘Robertsonian fan’. This view of the situation in Ethiopian *Acomys* I. Geoffroy, 1838 is based on standard chromosomal morphology that reveals a constant FN (68) and needs further investigation of chromosome homology by differential staining and/or molecular cytogenetic techniques as well as further molecular phylogenetic analysis.

## Introduction

Karyotypic studies on mammals of Ethiopia play an important role in species identification and in the interpretation of phylogenies of taxonomic groups (Lavrenchenko 2009). This in particular concerns the rodent taxa where karyotypic data constitute a powerful tool discriminating for an inventory of species diversity. Although the spiny mice of the widespread Afro-Asian genus *Acomys* I. Geoffroy, 1838 were in the focus of cytogenetic interests since the very beginning of current chromosome preparation era, i.e. the use of colchicine/hypotonic method (Wahrman and Zahavi 1953), the karyotype description of existing taxa in this group is far from complete. New data on karyotypic evolution are coming from the studies based on the chromosome banding techniques (Giagia-Athanasopoulou et al. 2011), and even the initial descriptions of chromosome numbers and morphology may be informative due to revealed wide chromosome variability and corresponding expectations of multiple chromosomal races or/and sibling species over wide generic distributional range (Volobouev et al. 1991).

It became clear from early descriptions of the Middle East populations that a special type of chromosomal variation, known as Robertsonian translocations, is to be regarded as the principal way of karyotypic evolution both between and within *Acomys* taxa. The differences in diploid numbers that was defined initially in representatives of *Acomys cahirinus* (É. Geoffroy, 1803) and *Acomys russatus* (Wagner, 1840) from Israel (2n=38 and 66, respectively), have shown almost exactly the 2n limits in the whole genus (Wahrman and Zahavi 1953). Further discoveries revealed even lower chromosome number (2n=36) in populations of *Acomys cahirinus* in the southern Sinai and had led to the discovery of a hybridization zone between the two intraspecific karyotypic forms (2n=36, 38) differed by a single whole-arm Robertsonian translocation (Wahrman and Goitein 1972). Data collected from species and populations of African *Acomys* since Matthey’s pioneer studies (Matthey 1956) up to now and in other parts of the generic range (for cytotaxonomic survey see Macholán et al. 1995, Volobouev et al. 1996, Zima et al. 1999, Dobigny et al. 2001, 2002, Granjon and Dobigny 2003, Corti et al. 2005, Sözen et al. 2008) show that diploid numbers for this genus vary from 2n=36 to 2n=68 via a series of intermediate 2n and the karyotypic constitution may be transformed from a totally acrocentric (2n=68) to almost fully metacentric (2n=36) complement.

The variation in chromosome arm numbers (the Fundamental Number, otherwise FN) is relatively low, from 66 to 78 (Fadda et al. 2001). The presence of FN numbers higher than 68 indicate that structural chromosome variations other than Robertsonian rearrangements, such as pericentric inversions and heterochromatin additions or deletions, occur in *Acomys* karyotypes.

In Ethiopia, only karyotypes with the extremal 2n values were previously found which have been reported for the two species inhabited the Lower Omo River Valley in the very south (2n=36 in *Acomys percivali* Dollman, 1911 and 2n=60 in *Acomys wilsoni* Thomas, 1892, according to Matthey 1968) and two species from the Rift Valley in the centre of the country (2n=36 in *Acomys cahirinus* and 2n=68 in *Acomys* sp., following Sokolov et al. 1993). Most close findings from Tanzania (Corti et al. 2005) show three different karyotypes with 2 nranged from 36 for *Acomys ignitus* Dollman, 1910 to 60 and 62 for *Acomys spinosissimus* Peters, 1852 and *Acomys wilsoni*, respectively. The different karyotype characteristics which were reported for the last taxon (2n=62 versus 60) were not considered in that publication.

In this communication, three new karyotypes of *Acomys* are presented which adds substantially to the 2n range known for taxa inhabiting this country (40, 44 and 52 versus 36 and 60, 68) and thus extends the series of Robertsonian transformations, known as a ‘Robertsonian fan’, performed in the related karyotypes. This study covers also three new geographic parts of Ethiopia where the spiny mice were never examined.

## Materials and methods

Animals were collected during the 2008 and 2010 field seasons in the course of the Joint EthioRussian Biological Expedition (JERBE). Chromosome preparations were obtained from five *Acomys* specimens collected in two lowland regions on opposite sides of the Rift Valley. Among them, two specimens of both sexes were from a geographic site in the east (Babille Elephant Sanctuary, 9.0601°N; 42.2699°E, 1200 m a.s.l.) and three specimens from two sites of the Alatish National Park, north-western Ethiopia (Amjale, 12.3875°N; 35.7309°E, 533 m a.s.l., 2 males, and Bermil, 12.4958°N; 35.6382°E, 575 m a.s.l., 1 female).

For the karyotype analysis, bone marrow cell suspensions were prepared according to the standard colchicine, hypotonic solution and air-dried technique (Ford and Hamerton 1956) and stained on microscopic slides with Giemsa; each individual was preserved for further molecular analyses, following to a current research trends (Bulatova et al. 2009, Lavrenchenko 2009). Collected specimens were not specifically identified in the field, but deposited for further morphological or DNA examination.

## Results

Three different karyotypes are found which characterize the Ethiopian *Acomys* of lowland savanna from three geographic samples in two different parts of the country. A higher chromosome number (2n=52) is detected in a male from Amjale. Its chromosome set contains lesser number of metacentric pairs than acrocentric ones (8 and 18, respectively; FN=68). Sex chromosomes cannot be reliably identified without differential staining, however, a larger acrocentric element with a distinguished short arm was recognized as the X chromosome, when one of the smaller acrocentrics may represent the Y chromosome. The autosomal arm number (FNa) characteristic for this karyotype is 66.

**Figure 1.**
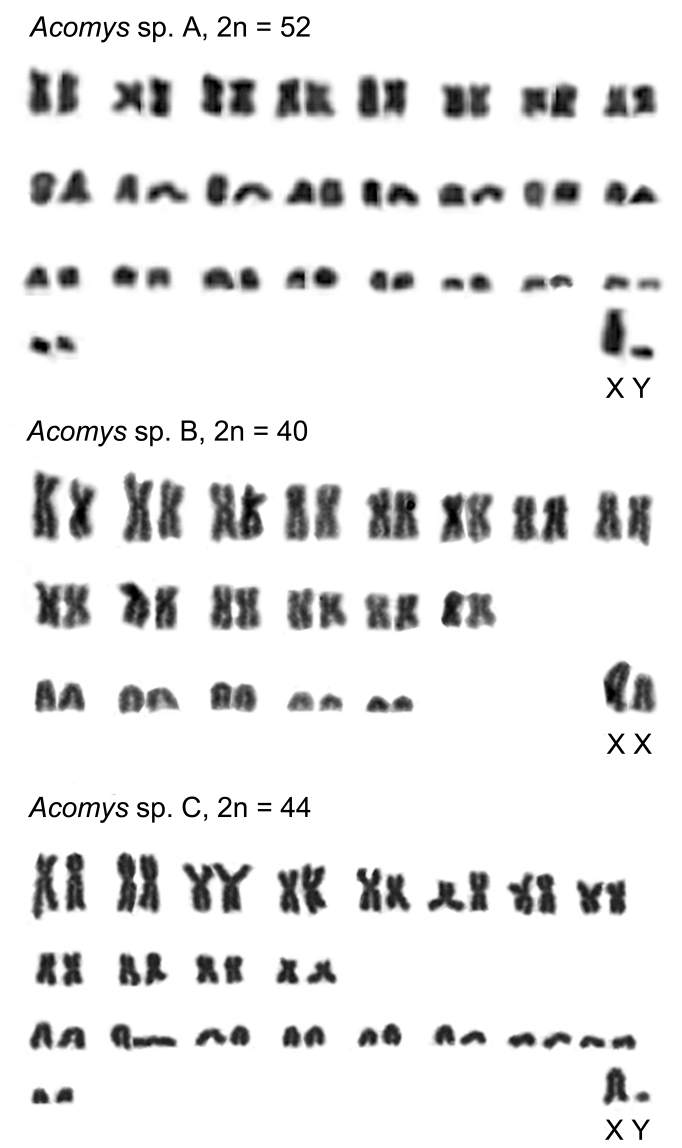
Karyograms of Ethiopian *Acomys* spp. A – Amjale, B – Bermil, C - Babille.

The lesser chromosome numbers are found in two other karyotypes (Figure 1). 2n=44 is characteristic for animals from the Babille Elephant Sanctuary. In this karyotype 12 metacentric and 10 acrocentric pairs are present (FN=68). The acrocentric chromosomes apparently include the XX pair in females and XY in males. NFa is 66 in this case. 2n=40 is reported from Bermil, and only 6 pairs of acrocentrics are identified in this karyotype (FN=68), one of which may be determined as the XX pair in a female studied, by analogy with the sex chromosomes of other *Acomys* species (Table 1). NFa is again 66.

## Discussion

Description of three new karyotypes of *Acomys* presented in this study indicates that there is more karyotype diversity in Ethiopian *Acomys* than it was thought previously. Three additional 2n numbers that were found (40, 44, and 52) fill in the series of 2nchanges where only minimal (36) and maximal (60, 68) limits were known until now (Table 1).

The common feature for five out of six karyotypes is the same number of chromosome arms (FN). The FN value is invariably 68 for full chromosome complements and 66 for the autosomes (FNa) in the karyotypes with various 2n which were attributed to at least five species, including *Acomys cahirinus* (2n=36) and four unidentified taxa, probably cryptic species - *Acomys* sp. (2n=68), *Acomys* sp. A (2n=52), *Acomys* sp. B (2n=40), and *Acomys* sp. C (2n=44) (Matthey 1968, Sokolov et al. 1993, our data).

Regarding this Ethiopian group, interrelations based on Robertsonian rearrangements could be suggested. It is well known that it is usually difficult to decide whether Robertsonian fusions or fissions occurred in each given group, and the interpretation of karyotype evolution depends upon the context of the comparative analysis. It is often a working hypothesis that a chromosome set with the most numbers of acrocentrics is the primitive one (2n=68 in the case of *Acomys*) and that karyotypic evolution should pass via successive fusions of any twin acrocentrics into one metacentric. In fact, due to the bi-directional Robertsonian process and probable multiple fusions of original acrocentrics, the chromosomal sibling species may exist that should confuse a hypothetic common chain of karyotypic changes from maximal to minimal 2n, or, in our case, from 2n=68 to 2n=36 via 2ns such as 52, 44 and 40. Indeed, a clear indication on Robertsonian, or centric fission has been obtained through the karyotype comparison between the continental (Turkey) *Acomys cilicicus* Spitzenberger, 1978 and Mediterranean sea island (Cyprus) spiny mouse, *Acomys nesiotes* (Bate, 1903), which differ in 2n as 36 and 38, respectively (Zima et al. 1999).

As for the 60-chromosome karyotype of *Acomys wilsoni*, one of the first reported from the Omo Valley by Matthey (1968), it is to be regarded as distinguished from the group mentioned above even on the level of routine chromosome data because it has a different FN=76 (FNa=74, see Table 1 and Introduction). In reference to modern chromosome data, it should be, however, questioned to what species does each of the two initial karyotype descriptions of south Ethiopian *Acomys* - *Acomys percivali* and *Acomys wilsoni* (sensu Matthey cit.) - belong. The first of the two was attributed to *Acomys cahirinus* after the work of Sokolov et al. (1993) as it has the same chromosome number, 2n=36 (see Table 1). Following recent karyotype descriptions from Tanzania, another chromosome numbers, 2n=62 and FNa=76, are attributed to *Acomys wilsoni* which is in this karyotypic form included into the current mammal species checklists (Fadda et al. 2001, Corti et al. 2005).

The spiny mice with 2n=60 were found in central and southern parts of Africa, but the karyotype peculiarities do not allow to consider them conspecific. Comparing to Ethiopian karyotype with FNa=74, two other species with the same 2n=60 show not only differences in FNa – 68 in *Acomys selousi* De Winton, 1896 and 70 in *Acomys spinosissimus* – but reveal serious difference in the morphological structure of X chromosome (metacentric with a heterochromatic arm in the first case and submetacentric in the second one). Moreover, they are characterized by the unique XO system in both sexes, accompanied with the atypical inter- and intraindividual autosome number variation (2n*=*58-62) (Barome et al. 2001, Fadda et al. 2001, Corti et al. 2005). In Ethiopian *Acomys* no other sex chromosome constitution than standard XX/XY is observed and there was no other X chromosome in their karyotypes than a moderate size acrocentric which is considered as the original type for the genus (Table 1).

There are still more examples of 2n similarity in the karyotypes of different *Acomys*. Three of four 2n values that were found in Ethiopia have been reported for *Acomys* from other territories, i.e. the first finding of 2n=68 from Burkina-Faso (see Sokolov et al. 1993) or 2n=44 from Mali (Dobigny et al. 2001), 2n=40 from Crete (Giagia-Athanasopoulou et al. 2011), and only 2n=52 is shown for this genus for the first time.

The 36-chromosome karyotype is most widely distributed in Mediterranean area and presented among African taxa. The same FN characteristics of the karyotypes with the same 2n may indicate the karyotypic clusters of common origin in the related groups. It is, however, interesting that preliminary data from mt-DNA analysis (Lavrenchenko, in prep.) focused on Ethiopian – i.e. East African – karyotypic forms that we described in this paper do not support their genetic affiliation with taxa from western parts of Africa with the same 2n=40 or 44 and FNa=66 (Nicolas et al. 2009). For analysis of geographically distant taxa, high-resolution chromosome banding should be used in addition to other methods. It was already shown that externally the ‘same’ 36-chromosome karyotypes could be formed via the complex Robertsonian arm fusions leading to partial, i.e.monobrachial homology between the variable metacentrics (Volobouev et al. 2007). If so, any finding of the 2n=36 karyotype does not seem to be a reason to attribute a corresponding taxon immutably to *Acomys cahirinus*.

Besides the Robertsonian changes occured generally in autosomes, heterochromatin variability exists regarding the X chromosome. To avoid probable variation in FN due to the presence/absence of a short heterochromatic arm in the X-chromosome, Macholán et al. (1995) suggested that the autosomal number, FNa, is better to be used for the karyotype comparison. Whether such kind of variation presents in the X with or without clear short arm as we observed in 3 karyotypes (Fig. 1) is to be evidenced by application of differential staining of heterochromatin.

As a result of this preliminary chromosome study, we may conclude that 3 new karyotypes (2n=40, 44, 52) are to be added to at least three karyotypic forms of Ethiopian *Acomys* (2n=36, 60, 68), described previously (Matthey 1968, Sokolov et al. 1993, our data). It follows from the comparison of data that five out of six karyotypes, except the one with 2n=60, may form a Robertsonian fan, the reliability of which is to be checked by further chromosome analysis of Ethiopian populations. And, finally, wide taxonomic expertise of the genus is needed based on the complex morphological, fine karyological and, in particular, molecular investigation of spiny mice inhabiting this country.

**Table 1.** Karyotypic data on *Acomys* collected from Ethiopia.

Species	2n	FN	FNa	X	Location	Reference
*Acomys wilsoni*	60	76	74	A	S Ethiopia: Omo Valley	Matthey 1968
*Acomys percivali	36	68	66	A	S Ethiopia: Omo Valley	Matthey 1968
*Acomys cahirinus*	36	68	66	A	S Ethiopia: Rift Valley (Konso; Arba-Minch)	Sokolov et al. 1993
*Acomys cahirinus*	36	68	66	A	Central Ethiopia: Rift Valley (2 sites along the middle Awash valley)	Sokolov et al. 1993
*Acomys* sp.	68	68	66	A	Central Ethiopia: Rift Valley (Koka, upper Awash valley)	Sokolov et al. 1993
*Acomys* sp. A	52	68	66	A	NW Ethiopia: Alatish National Park (Amjale)	This study
*Acomys* sp. B	40	68	66	A	NW Ethiopia: Alatish National Park (Bermil)	This study
*Acomys* sp. C	44	68	66	A	E Ethiopia: Babille	This study
Total: 6 or more species	36–68	68 (76)	66 (74)	A	Rift Valley and S, NW and E lowland Ethiopia	3 publications

* Referred to *Acomys cahirinus* in Sokolov et al. 1993.
